# Rapid Identification of Pathogens Causing Bloodstream Infections by Raman Spectroscopy and Raman Tweezers

**DOI:** 10.1128/spectrum.00028-23

**Published:** 2023-04-20

**Authors:** Katarina Rebrosova, Silvie Bernatová, Martin Šiler, Jan Mašek, Ota Samek, Jan Ježek, Martin Kizovsky, Veronika Holá, Pavel Zemanek, Filip Růžička

**Affiliations:** a Department of Microbiology, Faculty of Medicine of Masaryk University, St. Anne’s University Hospital, Brno, Czech Republic; b Institute of Scientific Instruments of the Czech Academy of Sciences, Brno, Czech Republic; c National Centre for Biomolecular Research, Faculty of Science, Masaryk University, Brno, Czech Republic; d Department of Plant Developmental Genetics, Institute of Biophysics, Academy of Sciences of the Czech Republic, Brno, Czech Republic; University Paris-Saclay, AP-HP Hôpital Antoine Béclère, Service de Microbiologie, Institute for Integrative Biology of the Cell (I2BC), CEA, CNRS

**Keywords:** Raman spectroscopy, bloodstream infections, diagnostics, sepsis, bacteria, *Candida*, Raman tweezers

## Abstract

The search for the “Holy Grail” in clinical diagnostic microbiology—a reliable, accurate, low-cost, real-time, easy-to-use method—has brought up several methods with the potential to meet these criteria. One is Raman spectroscopy, an optical, nondestructive method based on the inelastic scattering of monochromatic light. The current study focuses on the possible use of Raman spectroscopy for identifying microbes causing severe, often life-threatening bloodstream infections. We included 305 microbial strains of 28 species acting as causative agents of bloodstream infections. Raman spectroscopy identified the strains from grown colonies, with 2.8% and 7% incorrectly identified strains using the support vector machine algorithm based on centered and uncentred principal-component analyses, respectively. We combined Raman spectroscopy with optical tweezers to speed up the process and captured and analyzed microbes directly from spiked human serum. The pilot study suggests that it is possible to capture individual microbial cells from human serum and characterize them by Raman spectroscopy with notable differences among different species.

**IMPORTANCE** Bloodstream infections are among the most common causes of hospitalizations and are often life-threatening. To establish an effective therapy for a patient, the timely identification of the causative agent and characterization of its antimicrobial susceptibility and resistance profiles are essential. Therefore, our multidisciplinary team of microbiologists and physicists presents a method that reliably, rapidly, and inexpensively identifies pathogens causing bloodstream infections—Raman spectroscopy. We believe that it might become a valuable diagnostic tool in the future. Combined with optical trapping, it offers a new approach where the microorganisms are individually trapped in a noncontact way by optical tweezers and investigated by Raman spectroscopy directly in a liquid sample. Together with the automatic processing of measured Raman spectra and comparison with a database of microorganisms, it makes the whole identification process almost real time.

## INTRODUCTION

Even in the modern world, bloodstream infections are associated with high morbidity and mortality rates. Together with myocardial infarction, they are among the most common causes of hospitalizations. Patients with an increased risk of bloodstream infections include immunocompromised patients, patients with chronic infections, and elderly patients. Bloodstream infections frequently have a nosocomial origin: they are often associated with intravenous catheters. Another clinically significant and common type of bloodstream infection is infectious endocarditis, which occurs mainly in patients with preexisting heart disease, immunocompromised patients, or patients with artificial heart valves (1, 2).

Numerous bloodstream infections are caused by staphylococci (both coagulase-negative staphylococci and Staphylococcus aureus). However, the number of Gram-negative bacteria has been increasing, with Escherichia coli at the top. Also, other members of the family *Enterobacteriaceae* play a role. Other common causative agents of bloodstream infections include enterococci and yeasts of the genus *Candida* (mostly Candida albicans) (3–5). Due to increased travel to tropical countries, the role of parasites in bloodstream infections is growing. A separate category is viral sepsis, which is currently diagnosed very rarely. Therefore, estimates of their occurrence are very distorted (6).

Bloodstream infections often lead to sepsis, a life-threatening organ dysfunction caused by the deregulated response of a host organism to infection (7). Therefore, the treatment of bloodstream infections, including sepsis, must be intensive and complex. Its success depends on the timely identification of the causative agent and characterization of its antimicrobial susceptibility and resistance profiles. Choosing suitable antimicrobials should be individualized and often with consultation with specialists (8).

Gold-standard diagnostics of bloodstream infection rely on blood culture using automated instruments. Routinely, it is followed by the biochemical identification and phenotypic characterization of a susceptibility/resistance profile. The time necessary for the final identification and characterization of the antimicrobial susceptibility profile is from 48 h to several days (for slowly growing microbes) (9, 10). In the last decades, there have been many attempts to accelerate the process of clinical diagnostics by various methods. There are various systems to speed up the identification of causative agents, including PCR, *in situ* hybridization- or DNA microarray-based methods, the Accelerate Pheno system, and the Verigene system, which significantly shorten the time necessary for identification; however, they still require subcultivation. A breakthrough in this diagnostic process came with matrix-assisted laser desorption ionization–time of flight mass spectrometry (MALDI-TOF MS), an effective, quick, and relatively inexpensive method based on protein fingerprinting of microbes (11, 12). In addition, several protocols (including the ready-to-use Sepsityper kit) were designed to skip the subcultivation step and speed up the process even more. Before the analysis, microbes must be separated from the blood and the culture medium in blood culture bottles (to avoid interference with microbial spectra). The methods are based on centrifugation, the lysis of erythrocytes, and/or filtration; most include protein extraction (13–18). Several other methods were designed to obtain results even faster and skip the subcultivation step, which work directly with whole blood. These include, but are not limited to, multiplex real-time PCR kits (the Magicplex sepsis real-time test [Seegene]), the combination of PCR and magnetic resonance (T2Candida and T2Bacteria panels [T2Biosystem]), and metagenomic assays (e.g., the Karius next generation sequencing [NGS] plasma test, SepsiTest [Molzym], and iDTECT Dx blood [PathoQuest]) (19), but either they can detect a small spectrum of pathogens or their specificity is low. Therefore, none of the procedures is ideal for routine clinical use.

Raman spectroscopy belongs to optical methods with multiple applications in clinical medicine, such as measurements of inflammatory markers, including C-reactive protein (CRP) (20, 21); measurements of blood and urine chemicals (22); measurements of blood coagulation (23); and potential cancer diagnostics, even from human body fluids (23–26). In microbiology, numerous studies have shown the possibility of identifying microbes (27–38) and their virulence factors, including antibiotic resistance (39–41) and biofilm formation (42–44), by Raman spectroscopy and its variations. Raman tweezers, a combination of Raman spectroscopy and optical tweezers, allowed single-cell analyses of microbes in more depth by describing metabolic changes (45, 46). Raman tweezers can also identify microbes directly from liquid samples, including wastewater (47) and urine, in almost real time (<10 min) (48).

This study presents the possibility of differentiating microbes causing bloodstream infections by Raman spectroscopy. Furthermore, it describes a pilot study aiming to identify microbes directly from human serum. It compares the results obtained by Raman spectroscopy to the results obtained by MALDI-TOF MS.

## RESULTS

### Raman spectroscopy of colonies grown on Mueller-Hinton agar plates.

**FIG 1:**
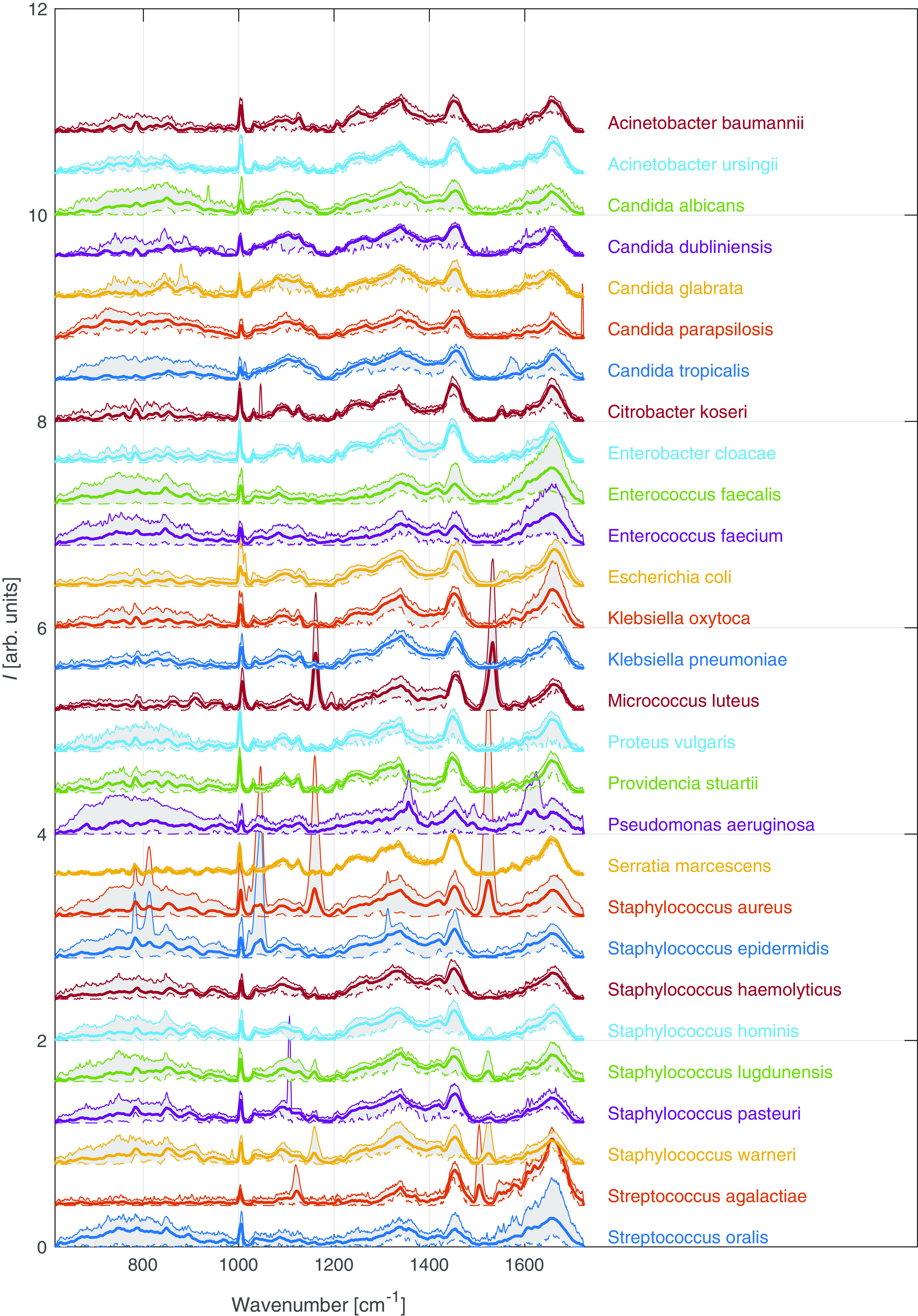
Averaged Raman spectra (bolded) of individual species, grown on agar plates, analyzed in this study after noise and fluorescence background removal normalized to the integral intensity. Gray areas indicate spectral intensity variations (solid curves, 5th percentile; dashed curves, 95th percentile) of individual Raman shifts. arb. units, arbitrary units.

We acquired Raman spectra from 305 strains belonging to 28 microbial species from colonies grown on Mueller-Hinton agar plates. Figure 1 presents the averaged spectra of individual species (bolded) and their variance limited by the 5th and 95th percentiles (colored patches).

For some species, we can see significant variances in Fig. 1. For most of the species, they are caused predominantly by the presence or absence of pigment production in the given strains. Pseudomonas aeruginosa’s peaks associated with pyoverdine include vibrations at 715, 830, 1,355, 1,488, and 1,611 cm^−1^. In staphylococcal species, we can detect carotenoid pigments visible at wavenumbers of 1,110, 1,160, and 1,525 cm^−1^, corresponding to C-C-(CH_3_), =C-C=, and -C=C vibrations (49). However, more factors contribute to the variance of spectra, including, but not limited to, virulence factors (e.g., the ability to form a biofilm [42], antimicrobial resistance [50, 51], and capsule in Klebsiella species [52]), the age and metabolic processes of the colony (53), and overall intraspecies biological variability.

**FIG 2:**
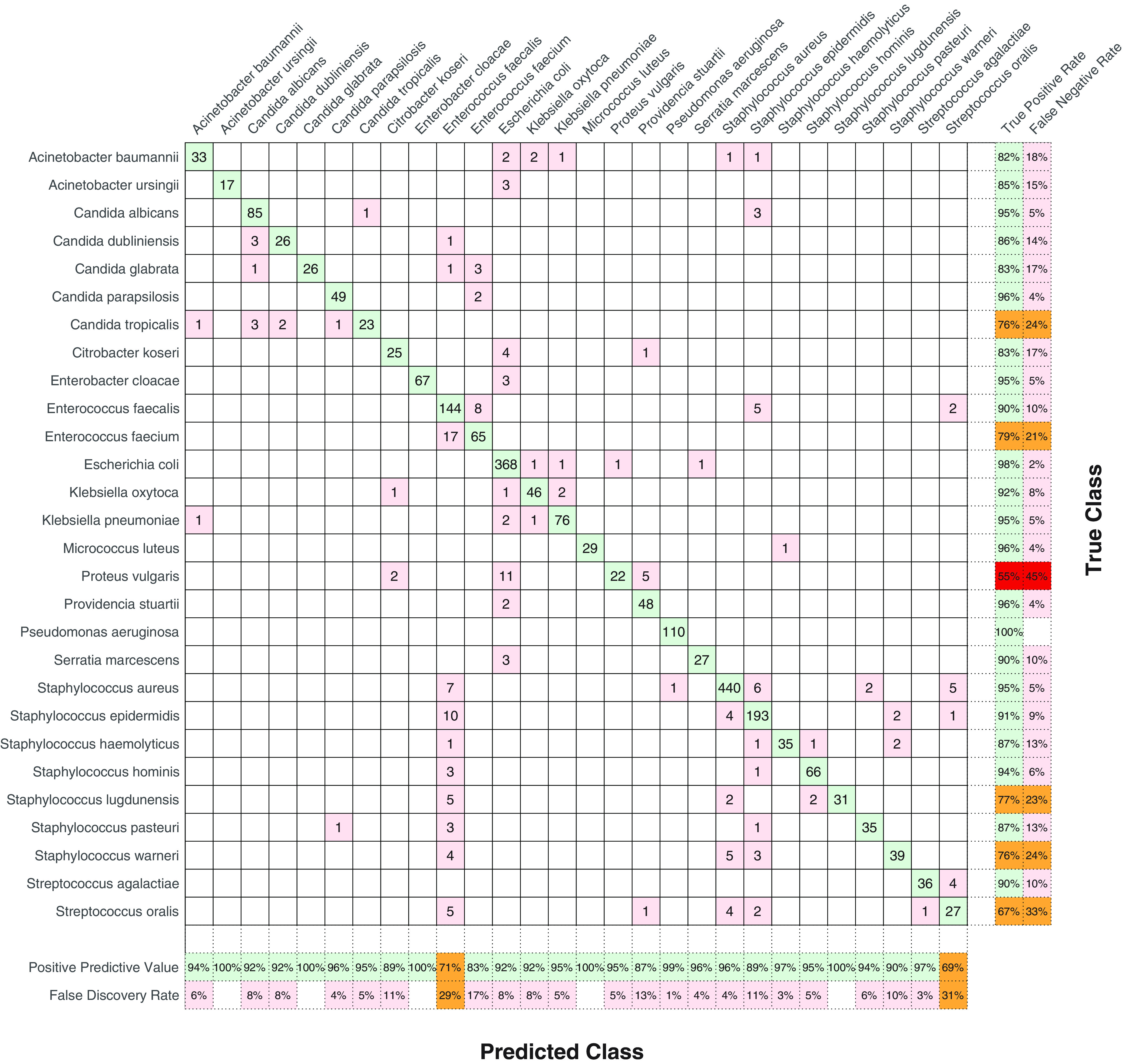
Confusion matrix showing the identifications of the analyzed strains (training set) by the SVM method. True class identifications (rows) correspond to the results of MALDI-TOF MS and biochemical methods; predicted class identifications correspond to the results predicted by Raman spectroscopy (SVM method). In the central part, color-coding indicates correctly identified spectra in green (diagonal) and incorrectly identified spectra in red (off-diagonal). In the marginal part, color-coding indicates successful identifications in green, partial failures in orange, and failures in red. The true-positive rate was calculated as the number of correctly identified strains/the number of correctly identified strains + the number of incorrectly identified strains. The false-negative rate was calculated as the number of incorrectly identified strains/the number of correctly strains + the number of incorrectly identified strains. The positive predictive value was calculated as the number of correctly predicted strains/the number of correctly and incorrectly predicted strains. The false discovery rate was calculated as the number of incorrectly predicted strains/the number of correctly and incorrectly predicted strains.

To distinguish among the species used, we compared two classification methods: one nearest neighbor (1NN) and support vector machine (SVM). Although the two classification methods have different principles, both approaches lead to similar results, with an accuracy of 93.5 to 93.8% for both 1NN and SVM using both centered and uncentered principal-component analyses (PCAs). The accuracy values were obtained by 5-fold cross-validation applied to the testing data. The results of cross-validation may be presented in the form of a confusion matrix (54), where the true class (rows of the matrix) corresponds to the identification of species based on MALDI-TOF MS and biochemical methods and the predicted class (columns) corresponds to the identification suggested by Raman spectroscopy. Correctly identified spectra (in green) are in the diagonal. Off-diagonal spectra (in red) correspond to incorrectly identified spectra (and their respective suggested classifications by Raman spectroscopy). The sensitivity of the method (true-positive rate [TPR]) and the false-negative rate are presented in the rightmost columns: they represent the relative counts of Raman spectra of given species that were incorrectly identified. The two bottom rows represent the positive predictive values (PPVs), relative counts of correctly identified spectra and spectra from different species falsely identified as a given species in each column, and the false discovery rate. In the ideal case, the PPV should be 100%. The results for the training set at the level of single spectra are illustrated in Fig. 2.

Subsequently, using the “semaphore” (for details, see Materials and Methods), we independently evaluated our method’s performance by classifying 71 testing strains with five possible outcomes. The green and orange species were either correctly or incorrectly identified as the true class identification (based on biochemical methods plus MALDI-TOF MS), with indistinguishable species being the fifth outcome. The results comparing the 1NN and SVM methods, which use features extracted by centered or uncentered PCAs, are summarized in Table 1. Each row represents one method, while columns represent the percentages of strains with each outcome. The critical part of classifier quality is suppressing misclassifications, i.e., the last two columns of Table 1, while maximizing the number of correctly identified strains with high confidence (third column). Furthermore, the ideal result would also minimize the number of indistinguishable strains in the fifth column. In this sense, we see that the performance of 1NN-based classifiers is much lower than that of the SVM, with around 12% wrongly classified strains and 13 to 17% indistinguishable strains. On the other hand, the SVM performs much better in the sense of wrongly classified strains, with only ~3% and 7% wrongly classified strains for centered and uncentered PCA feature extractions, respectively. However, as the centered PCA performs better on the wrongly classified data, it also contains a three-times-higher number of indistinguishable strains, which means that the number of correctly identified spectra is higher using the uncentered PCA. Therefore, we combined the results of the SVM classifications based on both PCA variants; i.e., we effectively obtained two classification results per spectrum. Consequently, we applied the semaphore to these values. The results of the “combined” PCA variants are shown in the bottom row of Table 1. The number of wrongly classified strains (4.2%) is only slightly higher than that in the case of the centered PCA, while the number of correctly classified strains, on the other hand, is close to results of the uncentered PCA.

**TABLE 1:** Semaphore-based results of performance testing of bacterial strain classifications using the 1NN and SVM methods combined with centered and uncentered PCA feature extractions

Method	PCA feature	% of strains with result				
		Green correct	Orange correct	Red	Orange wrong	Green wrong
1NN	Centered	67.6	4.2	16.9	4.2	7.0
	Uncentered	67.6	7.0	12.7	5.6	7.0
SVM	Centered	76.1	9.9	11.3	0.0	2.8
	Uncentered	81.7	7.0	4.2	2.8	4.2
Combined		80.3	7.0	8.5	0.0	4.2

**FIG 3:**
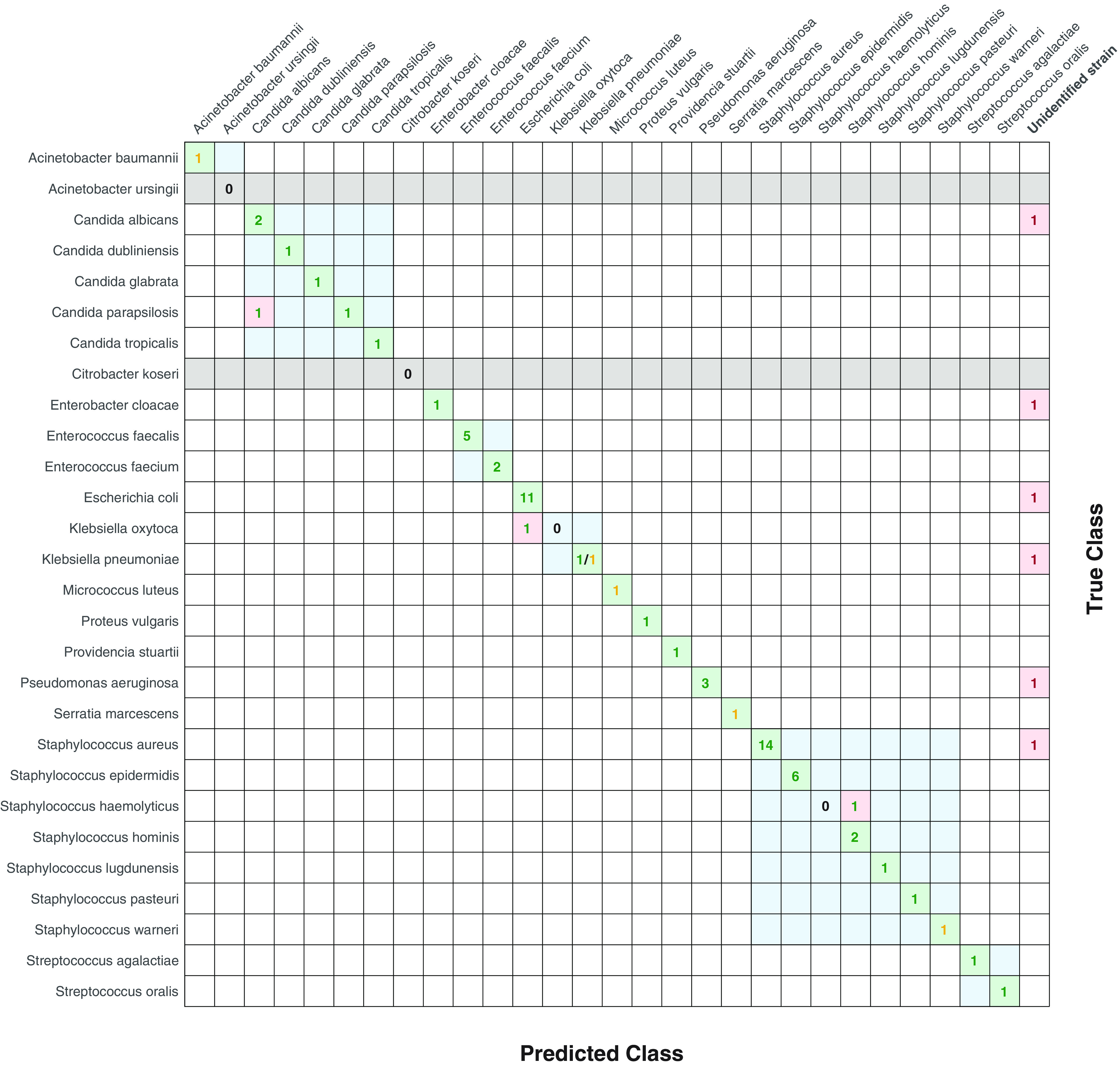
Confusion matrix showing the identifications of the test samples using spectral classification by the SVM method with the semaphore scheme (combining classifications based on the combination of centered and uncentered PCA feature extractions).

Finally, Fig. 3 depicts the results of model testing in the case of combined PCA variants in the form of a confusion matrix, as shown in Fig. 2. Numbers in the cells represent the numbers of strains belonging to a given species identified by biochemical methods plus MALDI-TOF MS (rows) and determined by Raman spectroscopy (columns). Moreover, if multiple species belong to one bacterial genus, these species are marked by light-blue squares. If no strain of a given species is available, the whole row is marked by a gray background. Green, orange, and red numbers correspond to the semaphore method. The correctly identified strains (on the main diagonal) are marked with a green background, while the incorrectly identified and indistinguishable strains are marked with a red cell background. We see the majority of strains located on the central diagonal, meaning that the correct identification is either green (high confidence) or orange (lower confidence). Only six strains are indistinguishable (last column), and three strains are wrongly identified. However, out of these three strains, two of those that we still identified were within the same genera (*Candida* and Staphylococcus). The remaining incorrectly classified strain is the one strain of Klebsiella oxytoca, which was identified as Escherichia coli.

Rows (true class) correspond to identifications by MALDI-TOF mass spectroscopy plus biochemical methods; columns (predicted class) correspond to identifications predicted by the SVM method. Numbers in the cells show correctly identified spectra on a green background (diagonal) and incorrectly identified spectra on a red background (off-diagonal). The colors of the numbers represent the confidence of the identification based on the semaphore scheme, with the indistinguishable samples (red semaphore) placed into the rightmost column. Moreover, gray rows depict the bacterial strains used in the training process, but no samples were present in the testing data set. Squares with a light-blue background depict bacterial species of the same genus.

### Analyses of spiked human sera using Raman tweezers.

**FIG 4:**
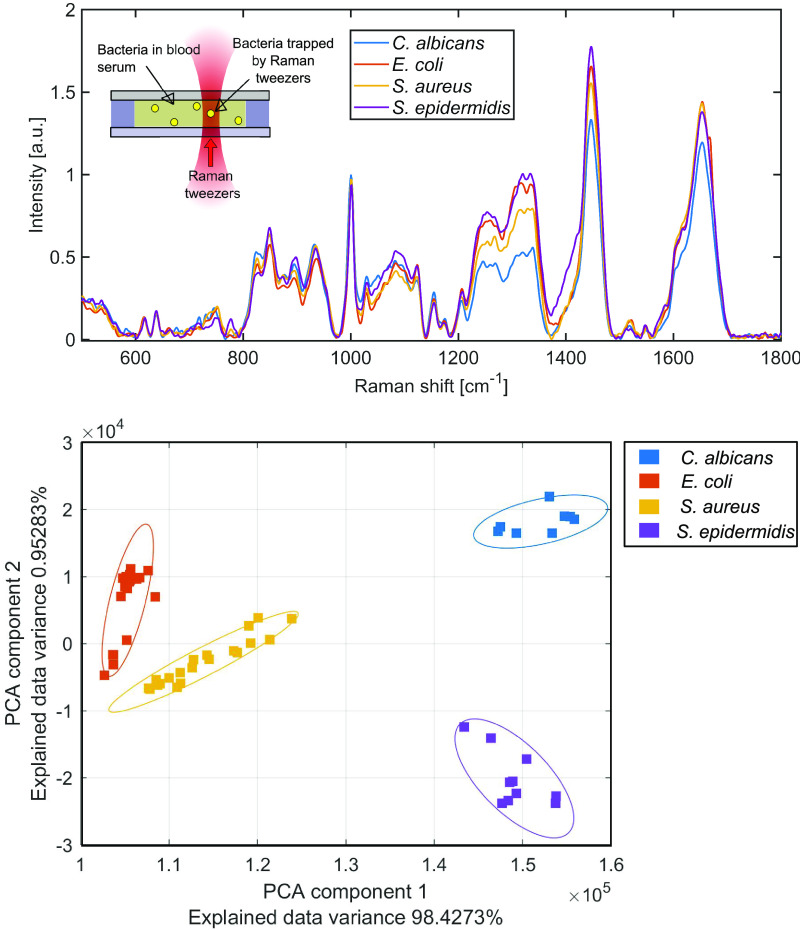
Average spectra of bacteria spiked into human serum and PCA plot differentiating spectra from the Raman tweezers system from individual cells in spiked human serum. Ellipses around the principal components were plotted using a Mahalanobis distance of 2.15 (corresponding to a 90% confidence interval). a.u., arbitrary units.

The cultivation step significantly slows the identification of pathogens in clinical diagnostic processes. Especially for life-threatening infections, the time to proper treatment is an essential component of treatment success. To shorten the time necessary for the identification of the causative agent, we performed a pilot study on spiked human sera using Raman tweezers, aiming to skip the cultivation step. The goal of this experiment was to test the feasibility of this application. In this case, optical tweezers attract microbes from the illuminated volume of the serum, catching them in an optical trap (up to 5 microbial cells dispersed in the serum). Since the laser beam is tightly focused, microbial cells cover most of the volume from which Raman spectra are acquired. In this way, the signal of the surrounding serum is suppressed (39, 48). The averaged spectra of the analyzed species in the serum, together with the PCA results, are presented in Fig. 4. Both the averaged spectra and PCAs show marked differences among the tested species, which proves the potential for the use of Raman tweezers for the identification of microbes directly in human serum.

## DISCUSSION

Our study shows that Raman spectroscopy can differentiate among pathogens causing bloodstream infections. We achieved approximately 94% correctly identified strains within the training set. Using the SVM classification method on the testing set and the “semaphore” approach, ~3% and 7% of the strains were incorrectly classified, with 86% and 88.7% correctly identified strains for centered and uncentered PCA feature extractions, respectively. These results closely correspond to those of previous studies using Raman spectroscopy to identify microbes (27, 28, 30, 32, 35, 38, 48).

When training the artificial intelligence (AI) classifier, the most problematic species was Proteus vulgaris, the spectra of which were repeatedly classified as Escherichia coli, suggesting a high level of similarity in their Raman profiles. Unsatisfactory results were also obtained for Candida tropicalis (identified as other *Candida* species in some instances), Enterococcus faecium (multiple times identified as being closely related to Enterococcus faecalis multiple times), Staphylococcus warneri and Staphylococcus lugdunensis (identified as other staphylococcal species and Enterococcus faecalis in some instances), and Streptococcus oralis (identified as various staphylococcal species and Enterococcus faecalis in a few cases). This led to a relatively high false discovery rate for Enterococcus faecalis (29%). Although these isolated problems occurred in the training set, all strains belonging to the species mentioned above from the testing set were identified correctly.

For the species with considerable observed variance in pigment production reflected in Raman spectra, namely, P. aeruginosa, Micrococcus luteus, S. aureus, and S. lugdunensis, the problems occurred with only one strain of P. aeruginosa and several individual spectra of *S. lugdunensis*. The set of S. aureus strains included 13 nonpigmented strains, 9 slightly pigmented strains, and 38 pigmented strains. Although the peaks associated with carotenoids are very intense, the machine learning algorithms did not consider them as the base for identifying S. aureus, so we can presume that this variability is suppressed by the rest of the characteristic spectral features.

We can conclude that despite some problematic strains, Raman spectroscopy offers an elegant, rapid approach to identifying microbes. It is noninvasive and nondestructive. Sample preparation is easy and does not require expensive consumables. When analyzing microbial colonies, it is sufficient to use a grown culture directly on a suitable culture medium (including, but not limited to, Mueller-Hinton agar, blood agar, and R2A agar). Alternatively, the colonies/microbial pellets after centrifugation and similar samples can be transferred to any Raman-inactive substrate or Raman-grade substrate with a defined low-interference signal (e.g., CaF_2_ slides and aluminum, etc.). Sample preparation for Raman tweezers depends on the sample; in the case of complex samples, preprocessing steps might be necessary. However, when the object of interest is in a liquid medium without other particulate matter or the particular matter is well defined, the objects can be directly captured and analyzed. Based on the principle of Raman tweezers, objects in the liquid medium are attracted by Raman tweezers and captured and can be subsequently analyzed. This allows the analysis of microbes in very low concentrations: the analysis is performed with one to four cells captured at once, so when the concentration is low, it takes more time to capture the object from the sample. The capture of an object is visible by the change in the laser beam appearance.

Moreover, Raman spectroscopy has a broad spectrum of potential applications in clinical diagnostics, including the detection of inflammatory markers such as CRP (20); differentiation between cancer and noncancer cells by Raman spectroscopy, Raman tweezers, and surface-enhanced Raman scattering (24–26, 55); or measurement of blood chemicals and coagulation (22, 23). Raman spectroscopy and/or Raman tweezers were also successfully used to determine antibiotic susceptibility or resistance in microbes (35, 39, 41, 56–58) and to detect other virulence factors, including the ability to form biofilms on solid surfaces (42–44). Biofilm formation is a common complication associated with bloodstream infections, especially in patients with intravenous catheters, implants, and other artificial devices in their bodies. Treatment of biofilm-associated infections poses a significant challenge since they can spread in the body and tend to be tolerant and/or resistant to (multiple) antimicrobials, and their proper management is difficult. Therefore, treatment needs to be accurately and promptly selected. It is necessary to identify a causative agent and its antimicrobial resistance profile to achieve this. Raman spectroscopy can identify microbes, their antimicrobial susceptibility versus resistance, and their ability to form a biofilm, proving its potential to become a valuable diagnostic tool in the future.

Using different sample preparation approaches, Raman spectroscopy has already been used to analyze microbes from various other clinical specimens, including ascitic fluid (59), sputum (60), saliva (61), urine (48, 62), and artificial bronchoalveolar lavage fluid (63). For the pilot experiments, serum was chosen as an example of a complex human-origin matrix without interfering particulate matter. We plan to adjust the procedure for whole human blood and blood cultures in the following steps. This will require preprocessing steps to eliminate human cells and other complex particulate matter that may be present.

Our pilot study implementing Raman tweezers to detect microbes in human serum suggests that Raman tweezers could detect the presence of microbes in human serum and characterize them, which could shorten the time necessary for identifying a pathogen to <10 min from the receipt of a clinical sample. This is in accordance with the results of our recent experiments with spiked human urine (48). Human urine is a good example of an easily accessible, complex human-origin matrix; however, its composition can significantly differ among individuals, which should be considered and addressed during analyses.

Based on the fundamental principle of Raman tweezers—it confines near its focus up to five microbial cells flowing in the serum (“attracting” microbes from the laser-illuminated volume into a limited trapping volume of a few cubic micrometers [therefore, the low numbers of microbes may slow the analysis but would not prevent it]) and acquires their Raman spectra (suppressing the signal from the surrounding serum by filling the trapping volume with microbes)—it might, after repeated measurements, give some indication of a possible mixed infection (the presence of more microbial species). Moreover, recent studies by Bernatová et al. (39), Pilát et al. (40), and Cui et al. (47) suggest the eligibility of Raman tweezers for the detection of antimicrobial resistance in microbes.

Altogether, this novel approach could significantly accelerate the identification of pathogens and their antimicrobial susceptibility/resistance profiles, leading to the timely and accurate treatment of a patient. Upon this pilot testing, we plan to perform a more extensive study to include more strains/species as well as more microbial species in the testing of the method.

Identifying and characterizing pathogens from tiny sample volumes rapidly and reliably are the crucial first steps in the diagnosis of microbial infections. Thus, we can envision the following wishful scenario in clinical practice. A sick person enters a hospital emergency ward with what appears to be an infection. Next, the doctor on duty collects a specimen (e.g., a blood sample) from them. Consequently, in an ideal case, the doctor could quickly and simply analyze the sample in the examination room. This analysis should give the doctor enough information for quantitative and conclusive pathogen identification. In this scenario, a point-of-care (POC) instrument that quickly identifies the pathogens in minutes is required. Thus, the clinician could prescribe tailored antibiotics, which improves the prognosis for a patient, shortens the time to proper treatment, and decreases problems with ever-increasing drug resistance. Since Raman spectroscopy and Raman tweezers have many possible applications in clinical medicine (as described above), they can provide a clinician with complementary information like the CRP level, the presence of inflammatory markers, and/or the presence of other conditions, including cancer. It could make the device suitable for complex clinical diagnostics.

Currently, the main limitations of Raman spectroscopy include the relatively high input costs necessary for purchasing the highly optimized instrumentation and the need for staff specifically trained in the acquisition of Raman spectra. Since microbial samples are heterogeneous, and variability can be expected, manual measurements are necessary at the current stage. They involve finding the object of interest (microbial colony/microbial cells), focusing on the sample, and analysis (preferably more measurements/strain). The training necessary for personnel should include mostly the finding and focusing phases, as they are essential for the outcome. The analysis can be performed with the same (or very similar) settings for most of the microbial samples of the same type (e.g., colonies or pellets, etc.), with some basic adjustments for specific situations. Therefore, we believe that the necessary training would not be more complicated than the training required for MALDI-TOF MS analyses.

Specially engineered portable systems could overcome these limitations of POC diagnostics in the future. Also, the absence of a large-scale database of microbial Raman fingerprints poses a problem for potential future clinical use. This is partially covered by our current (305 strains/28 species) and previous (27, 28, 48) work as well as the work of other groups in this field (32, 34–37, 64–69).

Beyond the limitations mentioned above, Raman spectroscopy has numerous advantages, proving its high potential to become a clinical diagnostic tool. It is noninvasive and nondestructive, so the samples (cells) can be used for further/complementary testing. It has a broad spectrum of applications across scientific fields to be used for numerous purposes in clinical diagnostics. It does not require any expensive consumables. Sample preparation is easy and quick. Therefore, we believe that Raman spectroscopy could become a valuable diagnostic tool in the future, significantly improving the management of infections and helping to reduce the speed of the ever-growing propagation of antimicrobial resistance. This would save the lives of patients as well as save costs for the management of infectious diseases.

### Conclusion.

The current study on 305 microbial strains belonging to 28 microbial species shows that Raman spectroscopy can reliably differentiate among microbes causing bloodstream infections, with only 2.8 to 7% incorrectly identified strains depending on the approach used. Moreover, the pilot study based on Raman tweezers suggests that it is possible to locate microbes directly in human serum; their Raman fingerprints are distinguishable among each other and the serum itself. Therefore, we believe that Raman spectroscopy and Raman-based techniques (Raman tweezers) could significantly contribute to the development of rapid approaches for the diagnosis and management of infectious diseases.

## MATERIALS AND METHODS

### Raman spectroscopy of colonies grown on Mueller-Hinton agar plates.

**(i) Sample preparation.** We measured 3,127 Raman spectra originating from 305 microbial strains of 28 species of 14 genera (see Table 2 for a detailed summary). The numbers of individual strains per species were based on the frequency of occurrence of individual species in positive blood cultures (3–5), and the number of included species outnumbers those in most of the common commercial tests for bloodstream infections.

**TABLE 2:** List of included species indicating the numbers of strains used, their division between the training and testing data sets for machine learning methods, and the included reference strains

Species	No. of strains	No. of strains used for training/testing	Reference strain(s)^*a*^
Acinetobacter baumannii	5	4/1	
Acinetobacter ursingii	2	2/0	
Candida albicans	12	9/3	CCM 8261, NCYC 1467
Candida dubliniensis	4	3/1	
Candida glabrata	4	3/1	
Candida parapsilosis	7	5/2	
Candida tropicalis	4	3/1	
Citrobacter koseri	3	3/0	
Enterobacter cloacae complex	9	7/2	
Enterococcus faecalis	21	16/5	CCM 5530, CCM 4224
Enterococcus faecium	10	8/2	
Escherichia coli	49	37/12	CCM 3954, CCM 3988
Klebsiella oxytoca	6	5/1	
Klebsiella pneumoniae	11	8/3	CCM 4895
Micrococcus luteus	4	3/1	
Proteus vulgaris	5	4/1	
Providencia stuartii	6	5/1	
Pseudomonas aeruginosa	15	11/4	CCM 1960, CCM 3955, CCM 7393
Serratia marcescens	4	3/1	
Staphylococcus aureus	60	45/15	CCM 3953, CCM 6188, CCM 4750, CCM 4223
Staphylococcus epidermidis	24	18/6	CCM 7221, CCM 4418
Staphylococcus haemolyticus	5	4/1	
Staphylococcus hominis	9	7/2	
Staphylococcus lugdunensis	5	4/1	CCM 4068
Staphylococcus pasteuri	5	4/1	
Staphylococcus warneri	6	5/1	CCM 2731
Streptococcus agalactiae	5	4/1	
Streptococcus oralis	5	4/1	

a CCM, Czech Collection of Microorganisms (Czech Republic); NCYC, National Collection of Yeast Cultures (UK).

Most of the strains were isolated from blood cultures of patients hospitalized at St. Anne’s University Hospital, Brno, Czech Republic, and stored in the Culture Collection of the Department of Microbiology, St. Anne’s University Hospital, Brno. The set also included 17 reference strains from the Czech Collection of Microorganisms (CCM) (Czech Republic) and 1 reference strain from the National Collection of Yeast Cultures (NCYC) (United Kingdom) (Table 2). Three Staphylococcus lugdunensis strains and one *S. warneri* strain included in the study were kindly provided by Petr Petráš from the National Reference Laboratory for Staphylococci in Prague, Czech Republic.

The strains were identified by biochemical methods and MALDI-TOF MS using the extended direct transfer method according to the manufacturer’s instructions (MALDI BioTyper protocol guide, 70). Single colonies were applied as a thin film onto the MALDI 96-target plate (Bruker Daltonik, Germany). Dried samples were overlaid with 1 μL of the matrix solution, a saturated α-cyano-4-hydroxycinnamic acid (Bruker Daltonik, Germany) solution in acetonitrile-water-trifluoroacetic acid (50:47.5:2.5, vol/vol), and allowed to dry before testing. Time of flight MS measurements were carried out using a MALDI BioTyper system (Bruker Daltonik, Germany) with FlexControl 3.4 software (Bruker Daltonik). Mass spectra were processed using BioTyper 3.1 software (Bruker Daltonik, Germany). All strains were identified to the species level with cutoff scores of ≥2.000.

The strains were stored at −70°C, thawed before the experiment, and inoculated onto Mueller-Hinton agar plates (Oxoid, Basingstoke, United Kingdom) or blood agar with 5% sheep erythrocytes (streptococci), which do not have a significant influence on Raman microbial fingerprints (71).

**(ii) Experimental setup.** We acquired Raman spectra from colonies grown on Mueller-Hinton agar plates after 24 h of cultivation at 37°C using a commercial Raman spectrometer (inVia Raman spectrometer; Renishaw PLC, Wotton-under-Edge, UK) equipped with a Leica N Plan EPI microscope objective (50×, numerical aperture of 0.75, working distance of 0.5 mm, and laser spot area of approximately 2 μm by 10 μm; Wetzlar, Germany). Spectral acquisition was performed using a 785-nm single-mode diode laser. The spectra were acquired in the range of 614 to 1,724 cm^−1^ (1,015 points) for 10 s.

We conducted at least 10 measurements of each microbial strain from 3 different colonies at a minimum, aiming the laser beam on the colonial surface. Before each spectral acquisition, the laser was refocused onto a colony surface, ensuring that the collected signal originated within the focal depth of the laser excitation and imaging optics. Considering the above-described geometry ensuring the collection of the Raman signal over an axial range of about 8 μm, we can neglect the contribution of the cultivation medium to the Raman spectra (71).

Measurements and sample preparations were performed in the same way on all measurement days. The high reproducibility of microbial Raman fingerprints acquired in this way was validated in our previous work (71).

**(iii) Data analysis.** We processed all acquired spectra using custom-written routines implemented in MATLAB software (MathWorks, Natick, MA, USA) (27, 28, 48). In comparison to our previous work, we modified the data processing and training of our classification algorithms for better correspondence to the clinical praxis. As we usually measure 10 Raman spectra per microbial strain, we treat this set of spectra as one “unit.” Therefore, for the training and testing of our classification models, we split the measured data set into two groups: training and testing. The training data set consists of approximately 75% of the measured strains. The measured samples (strains) belonging to one species were randomly separated, with three-quarters (rounded up) being assigned to the training data set and the remaining ones being assigned to the testing data set. In cases where there were only 1 to 3 strains for the given species (i.e., Acinetobacter ursingii and Citrobacter koseri), all spectra were assigned to the training data set. Table 2 also summarizes the number of strains used for training and testing in the third column. Furthermore, we did not consider any “outlier or other” data as, to our knowledge, we consider here the most significant microbial species occurring in clinical blood culture samples available in the mild-temperature region.

The Raman spectra in both groups were preprocessed by using the same procedure. Poisson photon detection noise was removed using the Savitzky-Golay filter (2nd order, with a width of 7 points). The high fluorescence background present in microbial spectra was removed by a rolling-circle filter (RCF) (10 passes, ~500 cm^−1^) (72). The spectra were normalized so that the total summed intensity equals 1.

### Training the AI classifier.

For the extraction of the main spectral features, an aid for the identification of microbial strains, we employed centered and uncentered principal-component analysis (PCA) variants. The extracted features (PCA scores) were used as the inputs for the one-nearest-neighbor (1NN) and support vector machine (SVM) classification methods. The 1NN method is based on identifying the closest data point of the known class (73), and the SVM constructs a high-dimensional border between subregions corresponding to a given class of data (74). As the numbers of spectra in each sample may vary, these classifiers were trained on features belonging to the single-spectrum input only. The optimal number of features for classification was selected upon an overall accuracy based on a 5-fold cross-validation scheme.

### Testing the quality of the classifications.

However, for testing the classification performance, we used the testing data set consisting of 741 spectra belonging to 71 strains that were not used for training our classifier. As each sample consists of multiple spectra, the classifiers are trained to provide results based on one spectrum only; we introduce a “semaphore” scheme to evaluate the testing results. The “semaphore-like” colors green, orange, and red represent the confidences of species classifications of high-confidence-level, low-confidence-level, and indistinguishable species. For this, we introduce the following set of rules: (i) at least 50% of the spectra must be classified as a single species, or the sample is indistinguishable; (ii) if the number of spectra belonging to the most probable species is at least three times the number of spectra of the second most probable species, the result is obtained with a high confidence level, i.e., green; (iii) if the number of spectra belonging to the most probable species is at least two times the number of spectra of the second most probable species, the result is obtained with a lower confidence level, i.e., orange; and (iv) otherwise, the sample is indistinguishable (red).

For example, let us assume that we take 10 spectra to classify one sample. We will obtain results with a high confidence level if 8 to 10 spectra belong to one species but also if the most populous species contains 6 or 7 spectra, assuming that the second most populous species has just 2 spectra, and also if 5 spectra are classified as a single species while the remaining 5 spectra are split, each of which gives a different species. On the other hand, we will obtain a low level of confidence of the classification if spectra are divided into groups according to 7-3, 6-3-1, and 5-2-2-1. The whole process is summarized in Fig. S1 in the supplemental material.

### Analyses of spiked human sera using Raman tweezers.

**(i) Sample preparation.** We used pooled anonymized serum samples from the Department of Microbiology, St. Anne’s University Hospital, Brno, Czech Republic. First, aliquots of the serum samples (1 mL) were spiked with S. aureus CCM 3953, Staphylococcus epidermidis CCM 4418, E. coli CCM 3954, and Candida albicans CCM 8261 (one strain/aliquot) and mixed thoroughly for at least 1 min using a vortex mixer. Ten microliters of the suspension was transferred to a glass chamber (one coverslip separated by a 120-μm spacer from a 200-μm thin CaF_2_ slide). This setup allowed us to obtain approximately 50 to 70 cells within the microscope field of view (approximately 100 μm by 100 μm).

**(ii) Experimental setup.** Freely moving microbial cells in spiked human serum were trapped by using a custom-built experimental Raman tweezers system: a combination of a Raman microspectrometer and optical tweezers using the same laser for trapping and Raman scattering (785 nm, catalog number TEC-510-0785-1000; Sacher, Marburg, Germany). This experimental setup achieves a full lateral width of the excitation region of ~0.8 μm. Therefore, only a few cells can be trapped and analyzed simultaneously (one to four cells), and the background (serum) signal is almost suppressed. Trapped cells were exposed to the laser beam only during spectral acquisition (50 s), and the laser was then blocked. Afterward, different cells were trapped in a new location within the sample, and we acquired their Raman spectra. Further details on the experimental setup and the procedure can be found in recent articles by Bernatová et al. (39) and Rebrošová et al. (48).

**(iii) Data analysis.** In the case of Raman tweezers experiments, the strong Raman signal of the medium (human serum) was suppressed according to the essential principle of optical tweezers: confining microbial cells in a focal volume of a laser beam, leading to the filling of almost the whole Raman scattering area by microbial cells. The acquired Raman spectra were processed using the Savitzky-Golay filter (2nd order, with a width of 7 points) and an advanced rolling-filter background removal routine (10 passes, with a 600-pixel circle radius of ~600 cm^−1^). The data were further analyzed using custom-written routines in MATLAB software (MathWorks, USA) using an uncentred PCA. PCA was also used to present results from Raman tweezers-based experiments. Ellipses around principal components were plotted using a Mahalanobis distance of 2.15 (90% confidence interval, assuming a Gaussian distribution of the data points).

### Data availability.

Raw data are available at https://osf.io/6t5r4/?view_only=3ec09e05ff5145639e1079ee772450a5 (OSF, USA).
