# Supplementary material for: Rapid Identification of Pathogens Causing Bloodstream Infections by Raman Spectroscopy and Raman Tweezers
Source: Microbiol Spectr. 2023 Apr 20;11(3):e00028-23. doi: 10.1128/spectrum.00028-23 (PMC10269886; doi:10.1128/spectrum.00028-23)
Supplement: Supplemental file 1 — Supplemental material. Download spectrum.00028-23-s0001.pdf, PDF file, 0.3 MB [file spectrum.00028-23-s0001.pdf]

Rapid identification of pathogens causing bloodstream infections by Raman spectroscopy and Raman tweezers

Katarina Rebrosova[1*], Silvie Bernatová[2], Martin Šiler[2], Jan Mašek[3,4], Ota Samek[2], Jan Ježek[2], Martin Kizovsky[2], Veronika Holá[1], Pavel Zemanek[2], Filip Růžička[1*]

[1] Department of Microbiology, Faculty of Medicine of Masaryk University and St. Anne's University Hospital, Pekařská 53, Brno 65691, Czech Republic

[2] Institute of Scientific Instruments of the Czech Academy of Sciences, v.v.i., Královopolská 147, Brno 61264, Czech Republic

[3] National Centre for Biomolecular Research, Faculty of Science, Masaryk University, Kamenice 753/5, Brno 625 00, Czech Republic

[4] Department of Plant Developmental Genetics, Institute of Biophysics, Academy of Sciences of the Czech Republic, Královopolská 135, Brno 612 65, Czech Republic

*Corresponding authors: k.mlynarikova@gmail.com (K.R.); fruzic@fnusa.cz (F.R.)

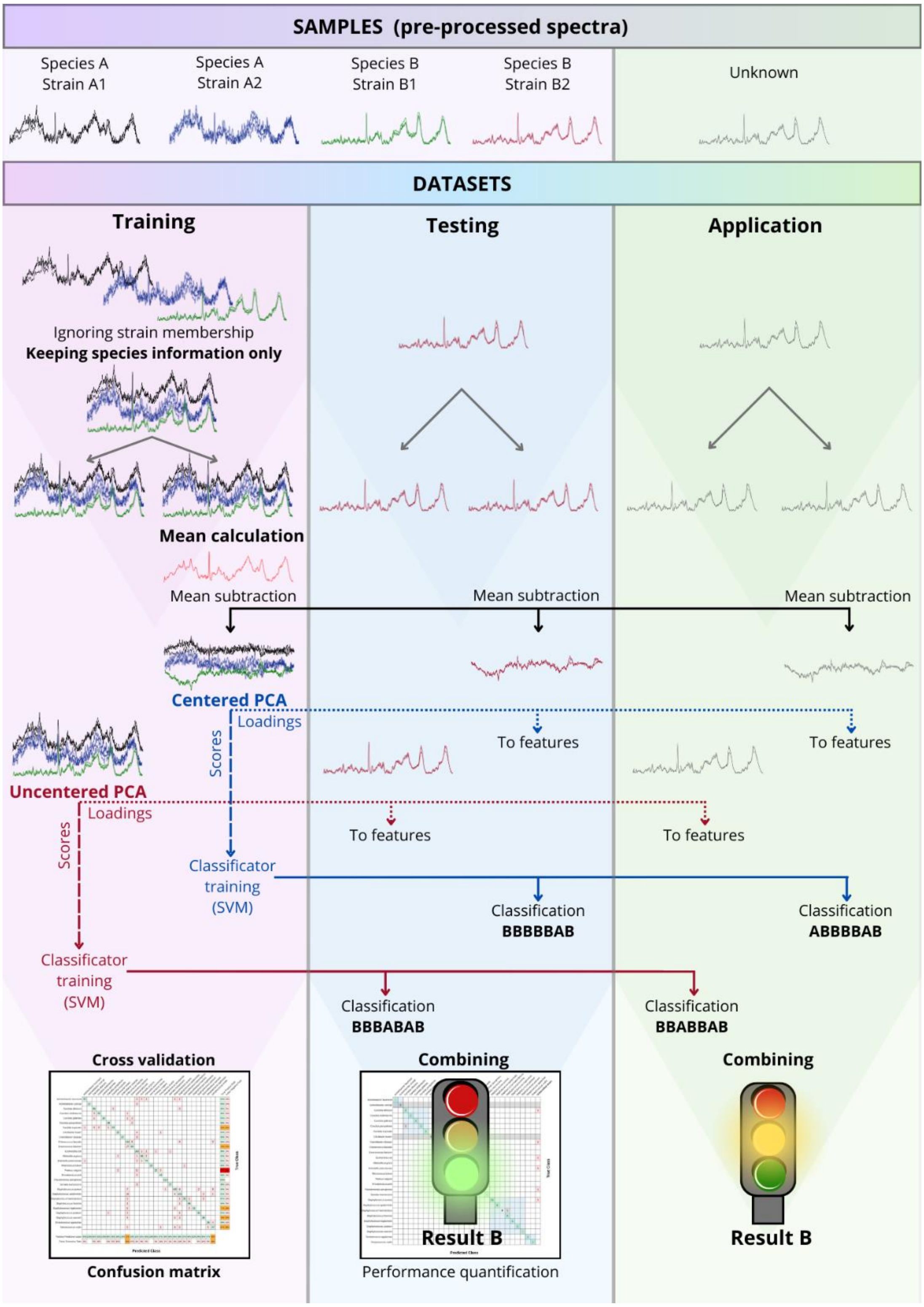

Figure S1. Workflow of the species classification process. The processed spectra (Savitzky-Golay filtered, fluorescence background removed and normalized), which had been independently identified by MALDI-TOF, are separated into training (~75% of strains) and testing groups (remaining strains). In the training group (the leftmost column), we employ

*Corresponding authors: k.mlynarikova@gmail.com (K.R.); fruzic@fnusa.cz (F.R.)

only the MALDI-TOF confirmed species and do not use strain sub-division. Centered and un-centered Principal Component Analysis (PCA) is applied independently on spectra in the training group giving two sets of PCA scores and loadings. PCA scores of both types represent features that are used to independently train the Support Vector Machines (SVM) classificators (the quality of training is evaluated by cross-validation confusion matrices, see Fig. 2). The testing data (spectra, the center column), are projected into the same subspace using mean, and loadings of the training data and the obtained features (scores) are used as inputs to the SVM classificators. The results of single spectra classifications are combined per strain, and the resulting species class is obtained by the semaphore scheme. The overall classification performance is summarized in Fig. 3 and Table 2 of the manuscript. The completely unknown sample consisting of ten spectra is typically processed similarly to testing data (see the rightmost column); for further details, see the main text.

*Corresponding authors: k.mlynarikova@gmail.com (K.R.); fruzic@fnusa.cz (F.R.)
